# Passion and Pacing in Endurance Performance

**DOI:** 10.3389/fphys.2017.00083

**Published:** 2017-02-20

**Authors:** Lieke Schiphof-Godart, Florentina J. Hettinga

**Affiliations:** ^1^Department of Human Movement Sciences, University Medical Centre Groningen, University of GroningenGroningen, Netherlands; ^2^Centre of Sport and Exercise Science, School of Biological Sciences, University of EssexColchester, UK

**Keywords:** regulation of exercise intensity, exercise behavior, psychobiology, overtraining, burnout

## Abstract

Endurance sports are booming, with sports passionates of varying skills and expertise battering city streets and back roads on their weekly or daily exercise rounds. The investments required for performing in endurance exercise are nevertheless considerable, and passion for their sport might explain the efforts endurance athletes are willing to make. Passion may be defined as a strong motivational force and as such might be related to the neurophysiological basis underlying the drive to exercise. A complex relationship between the brain and other systems is responsible for athletes' exercise behavior and thus performance in sports. We anticipate important consequences of athletes' short term choices, for example concerning risk taking actions, on long term outcomes, such as injuries, overtraining and burnout. We propose to consider athletes' type of passion, in combination with neurophysiological parameters, as an explanatory factor inunderstanding the apparent disparity in the regulation of exercise intensity during endurance sports. Previous research has demonstrated that athletes can be passionate toward their sport in either a harmonious or an obsessive way. Although both lead to considerable investments and therefore often to successful performances, obsessive passion may affect athlete well-being and performance on the long run, due to the corresponding inflexible exercise behavior. In this perspective we will thus examine the influence of passion in sport on athletes' short term and long term decision-making and exercise behavior, in particular related to the regulation of exercise intensity, and discuss the expected long term effects of both types of passion for sport.

## Introduction

Elite athletes continuously train and compete at the limits of their physiological and psychological capacities, and their training and competitive schedules are more demanding than ever (Schwellnus et al., 2016; Soligard et al., 2016). Passion for their sport might be the motivational force or “drive” providing these athletes with the required energy and determination to exercise, and might explain their willingness to endure discomfort (Curran et al., 2015) and even pain (Mauger, 2014), for the sake of sports performance (Vallerand et al., 2007). Pushing themselves to continuously sustain maximal effort over a long period of time might nevertheless entail several risks for passionate athletes, such as overtraining and injuries (Curran et al., 2015; Schwellnus et al., 2016; Soligard et al., 2016). We therefore propose that studying the nature of passion for sports might shed a light on athletes' exercise behavior (Vallerand, 2012) and subsequent risks.

In cyclic middle and long-distance exercise, athletes need to adequately estimate their physiological capacity and regulate exercise accordingly during training and races, in order to perform optimally (Foster et al., 2003; Abbiss and Laursen, 2008). As it represents a strong motivational force (Vallerand et al., 2008; Curran et al., 2015), passion might be a key factor in explaining their subsequent exercise behavior, both within a race and throughout the competitive season, derived from complex interactions between factors either promoting or reducing their drive to exercise (McCormick et al., 2015).

In this perspective, we will address athletes' exercise behavior and pacing by examining the impact of passion on the regulation of exercise intensity and exercise behavior from a psychological and a physiological angle. Adopting a psychophysiological approach to study the drive to exercise might elucidate athletes' pacing both on the short (during a single exercise bout or race) and long (a season or even an athletes' career) term and allow the examination of subsequent outcomes, such as athlete performance and well-being (McCormick et al., 2015).

## Passion in sports according to psychology

In the field of psychology, varying definitions of motivation have been proposed, most of which consider motivation as a combination of internal and/or external stimuli, pushing people to act or to react (Vallerand, 2012). In sports, motivation is used to describe the initiation, direction, intensity, and persistence of behavior (Vallerand, 2012), in short; the “drive” to exercise. Passion is defined as a “particularly strong motivation toward a self-defining activity,” and thus might be a useful construct to understand athletes' “drive” to exercise (Vallerand, 2008; Curran et al., 2015). As this drive seems imperative to sustain effort during endurance sports, notably at the elite level (Curran et al., 2015), understanding athletes' passion for sport might thus elucidate athletes' willingness to engage in demanding endurance exercise (Vallerand et al., 2003). The Dualistic Model of Passion proposed by Vallerand introduces passion as a key element in the long-term outcomes of engagement in activities (Vallerand, 2012; Bridekirk et al., 2016). By considering two distinct types of passion, namely harmonious and obsessive passion, this model explains apparent disparity in athletes' exercise behavior (Vallerand, 2012; Curran et al., 2015).

For athletes to develop a passion for their sport, they must highly value their activity, which distinguishes passion from both intrinsic and extrinsic motivation which can exist in different degrees from “not at all motivated” to “highly motivated” (Mageau et al., 2009). In accordance with Self-Determination Theory, different reasons for engaging in an activity might lead to different outcomes resulting from the two different types of passion (Ryan and Deci, 2000; Vallerand, 2012). Autonomous reasons, such as the inherent pleasure an activity brings, are known to lead to intrinsic motivation (Ryan and Deci, 2000). If the given activity becomes part of athlete's identity, a harmonious passion can result (Mageau and Vallerand, 2007). When athletes feel obliged to practice, or if the activity serves important compensatory or external functions, this will lead to a more extrinsic form of motivation, and, in time, can result in an obsessive passion (Mageau and Vallerand, 2007).

Passion and its resulting strong motivation are an essential prerequisite to attain and maintain the highest level in elite sport (Vallerand, 2012; Curran et al., 2015). A harmonious passion results from engaging repeatedly in an activity that provides athletes with positive emotional experiences, such as joy, feelings of mastery and high levels of concentration (Mageau et al., 2009; Bridekirk et al., 2016). Harmoniously passionate athletes feel free and autonomous in their choice to engage in their activity, and can maintain a healthy balance between their sport and other important life domains (Vallerand et al., 2008). Those with an obsessive passion for their sport perceive an unstoppable urge to practice, for example because their self-esteem relies on their athletic performance (Vallerand et al., 2007; Bridekirk et al., 2016).

Obsessive athletes feel they have no choice but to invest maximally in their activity: their passion has come to control their exercise behavior (Vallerand, 2012), which may lead to negative emotions (Curran et al., 2015; Bridekirk et al., 2016).

The nature of athletes' passion therefore influences decision making before and during exercise as well as throughout the competitive season (Mageau and Vallerand, 2007). Hence, understanding athletes' passion might (partially) elucidate their exercise behavior and the resulting benefits and drawbacks throughout the competitive season (Mageau and Vallerand, 2007; Vallerand, 2012).

## Passion in sports according to physiology: a neurophysiological perspective underlying the drive to exercise

The nature of athletes' passion, and thus of their drive to exercise, might represent one of the many factors influencing exercise behavior. In past research, the regulation of this drive to sustain exercise has been examined from a neurophysiological angle (Meeusen et al., 2006; Roelands et al., 2013). It has been found that neurotransmitters, such as dopamine, noradrenalin and serotonin, can maintain or decrease arousal and motivation to continue physical effort (Meeusen et al., 2006). Modifications in the release and re-uptake of neurotransmitters can influence the regulation of athletes' power output and central fatigue and hence influence exercise performance (Roelands et al., 2013).

Dopamine for example is known to play an important role in motivation, reward, attention, addiction, control of voluntary movement and locomotion (Meeusen et al., 2006). An increase in dopamine was found to enhance motivation toward rewarding goals in prolonged exercise, resulting in improved performance (Roelands et al., 2008a). Noradrenaline on the other hand is associated with the regulation of attention, arousal, anxiety, pain, mood, and depression (Meeusen et al., 2006). Increasing its concentrations resulted in decreased time trial performance (Roelands et al., 2008b). Although no effects of serotonin level manipulations on performance could be demonstrated, high serotonin levels appear to inhibit athletes' ability and motivation to perform an end sprint during exercise (Roelands et al., 2009).

In short, manipulations of brain dopamine or noradrenaline concentrations may delay fatigue, or on the contrary deteriorate performance in the heat (Roelands et al., 2015). Neurotransmitters thus might be considered as indispensable mediators between the drive to exercise and actual behavior, leading to adjustments in energy expenditure and effort exerted (Roelands et al., 2013).

## Regulation of exercise intensity and endurance performance

The goal-directed regulation of exercise intensity over an exercise bout in which athletes need to decide how and when to invest their energy has been defined as pacing (Smits et al., 2014) and its outcome can be measured as athletes' power output or velocity profile during exercise (de Koning et al., 1999; Foster et al., 2003). In order to perform optimally, athletes must choose the best pacing strategy for a given situation, based on their physiological and psychological capabilities (Baron et al., 2009). Fatigue, ambient conditions and manipulations of brain neurotransmitters clearly act on athletes' regulatory mechanisms and hence affect their exercise behavior (Roelands et al., 2013). The relationship between brain neurotransmitters and athletes' pacing behaviors seems to be mediated by the subjective interpretation of their physical state, also known as their rate of perceived exertion (RPE) (Borg, 1982; Marcora and Staiano, 2010). Interestingly, psychological factors implicated in athletes' endurance performance are thought to act through these same mechanisms. Both physiological and psychological factors act on motivation to continue or perception of effort and thus are important in the regulation of exercise intensity (Abbiss and Laursen, 2008).

In understanding exercise regulation, most recent theories share the consideration of an interaction between the physical work realized and the cognitive and psychological interpretation of the sensations this physical work causes (Abbiss et al., 2015). Millet, for example, propose the flush-model, which provides useful insight on how environmental conditions, such as sleep deprivation or nutritional strategies, may affect ultra-endurance performance (Millet, 2011). This holistic model incorporates ratings of perceived fatigue as a crucial element of the regulation of exercise intensity and provides insight in the complexity of exercise regulation (Millet, 2011).

Fatigue during exercise has been described as being composed of both a neurophysiological and a subjective component (Pereira et al., 2014; Enoka and Duchateau, 2016). Cognitive and psychological factors thus are considered to play a key role in the perception of fatigue, for example in the psychobiological model. (Marcora and Staiano, 2010; Noakes, 2012). It seemshat perception of effort or fatigue can be regarded as a source of information, used for regulating exercise intensity (Marcora and Staiano, 2010; Noakes, 2012). Maladaptive pacing strategies therefore may result from a misinterpretation of RPE or, for example, concentrating on the wrong stimuli. Due to the subjective element in the perception of effort and fatigue, athletes are at risk of either investing too much or insufficient energy during their race leading to submaximal performance (Foster et al., 1993; Baron et al., 2009). An optimal pacing strategy requires the continuous selection of the appropriate amount of effort to exert at any given moment (Baron et al., 2009), hence decision-making aspects are crucial in endurance exercise performance (Renfree et al., 2014; Smits et al., 2014; Micklewright et al., 2016). Recently, novel theoretical frameworks have been developed in order to explore decision-making and pacing.

Human-environment interactions seem crucial in understanding the regulation of exercise intensity in competitive situations. Internal factors, such as fatigue, and external factors, such as the behavior of an opponent, have indeed been shown to influence the drive to exercise, pacing and performance (Smits et al., 2014; Konings et al., 2016b). Athletes seem to adapt their behavior to the actions of their opponents, for providing athletes with an opponent or feedback has been shown to impact on their power output, in middle-distance and endurance exercise (Noorbergen et al., 2015; Konings et al., 2016a,b; Smits et al., 2016). Between competing athletes of comparable physical capacities, the drive to exercise and its resulting decision making might thus represent the difference between victory and defeat (McCormick et al., 2015).

Interestingly, the observed effects of several external manipulations (Konings et al., 2016b) have been similar to those of the previously described pharmacological (and thus internal) interventions (e.g., Meeusen et al., 2006; Roelands et al., 2013). This drive to exercise and its magnitude can thus be influenced by manipulation of various internal and external factors. One of these factors could be the nature and magnitude of an athlete's passion (Mageau and Vallerand, 2007; Vallerand, 2012), influencing pacing behavior during exercise for example by modifying the perception and prioritizing of different stimuli (Curran et al., 2015; McCormick et al., 2015; Bridekirk et al., 2016).

## Passion and pacing

Passion for sport is a strong motivational force, encouraging athletes to engage in strenuous exercise and pushing them to realize the countless training sessions and competitions needed to reach the top. Hence it might aid in enhancing athletic performance (Vallerand, 2012; McCormick et al., 2015).

Harmonious passion indeed leads to a strong drive to exercise, compatible with enhanced concentration, feelings of mastery and positive emotions during a race (Mageau and Vallerand, 2007; Vallerand, 2012). These positive emotions have been linked to the development of optimal pacing behavior in sports, as athletes can use and manage their emotions to guide them toward optimal performance (Baron et al., 2009), and to weigh their options wisely (Vallerand et al., 2008; Curran et al., 2015). Harmonious passion also protects against negative distraction, unrealistic goals and stress during exercise (Vallerand et al., 2007; Curran et al., 2015), and helps athletes to prioritize long term benefits over short term profits (Rip et al., 2006; Vallerand, 2012; Curran et al., 2015). As such, harmonious passion is believed crucial for attaining high levels of performance both on the short and the long term (Vallerand, 2012).

Although obsessive passion intuitively seems beneficial, it mostly leads to negative outcomes, both on the short as on the long term (Mageau and Vallerand, 2007; Curran et al., 2015). Due to its compulsive nature, this type of passion is related to negative affect, stress, anxiety and feelings of guilt during exercise (Vallerand, 2012; Curran et al., 2015; Bridekirk et al., 2016).

During a race, dealing with both negative emotions and the task at hand is mentally and emotionally exhausting, so obsessive passion decreases athletes' ability to concentrate and attain a state of “flow” (Bridekirk et al., 2016). Moreover, it hampers endurance performance by causing augmented feelings of exertion or erroneous interpretations of the athlete's capacities (Bridekirk et al., 2016). A strong obsession with their activity may thus render athletes incapable of regulating their efforts wisely (Curran et al., 2015; Bridekirk et al., 2016) and may result in risky, all-out pacing strategies and in the inability to accept defeat (Curran et al., 2015; Bridekirk et al., 2016). Obsessive athletes also might intentionally disregard the limits of their physical and mental capacities for the sake of a short term victory (Curran et al., 2015), hence disregarding rational and “safe” decisions that might prevent accidents, overtraining and overuse injuries (Rip et al., 2006).

Passion of an obsessive nature is therefore expected to prove particularly disadvantageous, considering the complex task of managing training and competition loads throughout a season (Bridekirk et al., 2016; Schwellnus et al., 2016; Soligard et al., 2016). This inadequate decision-making regarding exercise behavior might eventually lead to overuse injuries, overtraining syndrome and burnout in obsessive athletes (Vallerand, 2012; Curran et al., 2015).

## Further practical implications in sports: training adherence, burnout, and overtraining syndrome

The limiting factor for reaching and maintaining elite level in sports nowadays seems to be an athlete's capability to remain injury-free and to endure high training loads for a long period of time (Schwellnus et al., 2016). Current demands in elite sports enhance athletes' risk for insufficient physical and mental recovery (Meeusen et al., 2013). This may lead to athletes experiencing burnout or overtraining syndrome, resulting in physical and mental exhaustion and a considerably decreased drive to exercise (Curran et al., 2015). Several biological, neurochemical, and hormonal regulation mechanisms associated with the development of these symptoms have been identified (Meeusen et al., 2013). A major risk factor for injuries, overtraining and illness might be inadequate load management throughout the season (Schwellnus et al., 2016; Soligard et al., 2016), which in turn has been suggested to be affected by factors of various nature, such as fatigue, psychology, and metabolic and hormonal factors (Schwellnus et al., 2016; Soligard et al., 2016). Symptoms of athlete burnout for example include both emotional and physical exhaustion (Rip et al., 2006). Based on the above, we propose that athletes' passion and drive to exercise might lead to either adequate or inadequate pacing behavior and the subsequent effects on long term outcomes during an athlete's career. It was indeed demonstrated that measuring physical training load does not suffice in predicting or preventing long term injuries and neither do neurophysiological parameters alone (Meeusen et al., 2013; Schwellnus et al., 2016; Soligard et al., 2016).

Overtraining and burnout may be caused by an interaction of both physical and psychological factors. Indeed, modifications of neurotransmitters in the brain lead to an alteration in pacing and willingness to exercise but also result in both the physical and emotional exhaustion characteristic for athlete burnout (Roelands et al., 2013; Curran et al., 2015). In addition to these physiological parameters, psychological aspects, such as mood states, already have been identified as possible markers of overtraining and athlete burnout (Soligard et al., 2016). Indeed, perceived motivational climate as well as athletes' commitment and coping with adversity might increase their vulnerability to detrimental long term outcomes (McCormick et al., 2015; Schwellnus et al., 2016; Soligard et al., 2016). These factors interestingly all are closely related to the nature of athletes' passion for and drive to exercise (Vallerand, 2012; Curran et al., 2015; Bridekirk et al., 2016).

## Conclusion

Elite endurance athletes must display a considerable drive and thus a great passion for their sport in order to perform at elite level (Vallerand, 2012). Neurotransmitters have been identified as indispensable mediators between this drive to exercise and athletes' actual behavior. They inform athletes about their physical limits and the importance of continuing their effort, leading to adjustments in pacing strategies. In addition, external stimuli, such as opponents, could evoke similar responses by increasing the drive to exercise, hence affecting athletic decision-making. Passion for sport may come with a price for athletes obsessive about their sport.

We propose that the nature of elite athletes' passion for sport may provide useful insight in their decision making during exercise. It might affect pacing during a single exercise bout, but also influence exercise behavior during one or several years. Taking into account athletes' passion could therefore be a useful tool for adequate coaching and monitoring of athlete well-being. Although some athletes may be admirably capable of fitting their sport and high training loads into their ever-busy lives, obsessive athletes are expected to be extremely vulnerable to risks of injuries including overuse injuries, overtraining, burnout and drop out (Vallerand, 2012; Curran et al., 2015; Soligard et al., 2016).

## Author contributions

LS and FH contributed to conception and design of the work, drafted it and revised it critically for important intellectual content. Both authors have approved the final version of the manuscript, agree to be accountable for all aspects of the work in ensuring that questions related to the accuracy or integrity of any part of the work are appropriately investigated and resolved. All persons designated as authors qualify for authorship, and all those who qualify for authorship are listed.

### Conflict of interest statement

The authors declare that the research was conducted in the absence of any commercial or financial relationships that could be construed as a potential conflict of interest.
